# The Vital Role of Career Pathways in Nursing: A Key to Growth and Retention

**DOI:** 10.7759/cureus.38834

**Published:** 2023-05-10

**Authors:** Abdulqadir J Nashwan

**Affiliations:** 1 Nursing Department, Hamad Medical Corporation, Doha, QAT

**Keywords:** healthcare organizations, staff retention, professional development, nursing workforce, career pathway, nursing

## Abstract

This editorial highlights the importance of career pathways for nurses, emphasizing their role in fostering personal and professional growth, building a diverse and adaptable nursing workforce, and promoting staff retention. Healthcare organizations can empower nurses to reach their full potential and address the nursing shortage by offering a clear roadmap for advancement. The development and promotion of career pathways contribute to a stable and experienced workforce and ensure the delivery of high-quality patient care in today's complex healthcare environment. Prioritizing career pathways is crucial for nursing education, professional development, and long-term success in the healthcare sector.

## Editorial

The nursing profession has long been a cornerstone of our healthcare system, providing the essential care and expertise that patients need during critical moments in their lives [1]. As the demand for highly skilled nurses continues to grow, it is crucial that we consider the importance of career pathways for nurses and the impact they have on professional development and staff retention.

Career pathways for nurses are not merely a way to map out potential progressions; they also foster personal and professional growth [2]. By providing a clear and structured roadmap, nurses can visualize their long-term career goals, identify the necessary steps to achieve them, and make informed decisions about their professional development. This sense of purpose and direction empowers nurses to reach their full potential and enables them to adapt to the ever-evolving healthcare landscape.

Moreover, career pathways are essential to building a resilient and diverse nursing workforce [3]. When nurses are encouraged to explore different areas of practice, they can acquire a wide range of skills and knowledge, making them more versatile and valuable to their organizations. This adaptability is crucial in today's complex healthcare environment, where the challenges faced by nurses are as diverse as the patients they serve.

Investing in career pathways for nurses is also a strategic move for healthcare organizations, as it directly correlates with staff retention. By providing clear opportunities for growth and development, organizations demonstrate their commitment to nurturing the talents of their nursing staff. This support is crucial in maintaining employee satisfaction and engagement. A study by the American Association of Critical-Care Nurses (AACN) found that hospitals with professional development programs in place experienced a 34% decrease in nurse turnover rates [4]. The implementation of these programs not only reduces the financial burden of high turnover but also contributes to a stable and experienced workforce that can consistently deliver high-quality care.

There are several reputable programs available that support nurses in advancing their careers. One such example is the Magnet Recognition Program®, created by the American Nurses Credentialing Center (ANCC). This program promotes excellence in nursing practice and fosters professional growth within a supportive environment. The National Health Service (NHS) in the United Kingdom also offers initiatives like the Preceptorship Framework and the Clinical Academic Careers Framework to aid nurses in career advancement. Additionally, Hamad Medical Corporation (HMC) in Qatar, one of the largest healthcare providers in the Middle East, has implemented a career framework that provides structured support and guidance to nurses throughout the career planning process. This framework enables nurses to acquire new skills and achieve their professional goals.

Additionally, career pathways can help combat the nursing shortage [5]. As older nurses retire and the need for healthcare services increases, the gap between the number of nurses required and the number available will only grow wider. Healthcare organizations can attract new talent and retain their experienced staff by offering enticing career opportunities and promoting professional development.

In conclusion, the development and promotion of career pathways for nurses are vital for the nursing profession's growth and the healthcare system's stability. By recognizing and supporting nurses' career aspirations, healthcare organizations can foster a highly skilled, diverse, and motivated workforce better equipped to navigate the complex challenges of modern healthcare. To ensure the longevity and excellence of our nursing workforce, it is crucial that we prioritize career pathways as a key component of nursing education, professional development, and staff retention strategies.
